# Zinc oxide nanostructures enhanced photoluminescence by carbon-black nanoparticles in Moiré heterostructures

**DOI:** 10.1038/s41598-023-36847-1

**Published:** 2023-06-15

**Authors:** Chih-Chiang Wang, An-Ya Lo, Ming-Che Cheng, Yu-Sung Chang, Han-Chang Shih, Fuh-Sheng Shieu, He-Ting Tsai

**Affiliations:** 1 Department of Chemical and Materials Engineering, National Chin-Yi University of Technology, Taichung, 411030 Taiwan; 2 Department of Materials Science and Engineering, National Chung Hsing University, Taichung, 40227 Taiwan; 3 Department of Chemical Engineering and Materials Science, Chinese Culture University, Taipei, 11114 Taiwan; 4 Instrument Center, The Office of Research and Development, National Chung Hsing University, Taichung, 40227 Taiwan

**Keywords:** Materials science, Nanoscale materials, Nanoscience and technology, Nanoscale materials

## Abstract

ZnO/carbon-black heterostructures were synthesized using a sol–gel method and crystallized by annealing at 500 °C under 2 × 10^−2^ Torr for 10 min. The crystal structures and binding vibration modes were determined by XRD, HRTEM, and Raman spectrometry. Their surface morphologies were observed by FESEM. The Moiré pattern that is observed in the HRTEM images confirms that the carbon-black nanoparticles were covered by the ZnO crystals. Measurements of optical absorptance revealed that the optical band gap of the ZnO/carbon-black heterostructures increased from 2.33 to 2.98 eV as the carbon-black nanoparticle content increases from 0 to 8.33 × 10^−3^ mol owing to the Burstein–Moss effect. The photoluminescence intensities at the near-band edge and of the violet, and blue light were increased by factors about 68.3, 62.8, and 56.8, respectively, when the carbon-black contents is of the 2.03 × 10^−3^ mol. This work reveals that the proper carbon-black nanoparticle content involved increases the PL intensities of the ZnO crystals in the short wavelength regime, supporting their potential application in the light-emitting devices.

## Introduction

Zinc oxide is a promising material for use in the light-emitting devices^1^, photocatalysts^2^, gas sensors^3^, and solar cells^4^, owing to its n-type semiconducting property, wide band gap (3.3 eV)^5^, high exciton binding energy (60 meV)^5^, environmental friendliness^6^, low cost, and high physical and chemical stabilities^7^. Two methods that involve doping with elements Sb^8^, Ga^9^, Cu^10^, Gd^11^, and Li^12^, and heterostructures, such as RGO/ZnO^2^, Ag/ZnO^6^, ZnO/graphene^13^, Si/ZnO^14^, In_2_O_3_–ZnO^15^, and MoS_2_@ZnO^16^, can be used to modify and improve the emission of the light by ZnO. The most common means of synthesizing ZnO nanostructures include sol–gel^6^, thermal hydrothermal^17^, microwave hydrothermal^18^, thermally chemical vapor deposition (CVD)^8^, and the pulsed laser ablation (PLA)^19^ methods. As mentioned above, the sol–gel and thermal CVD approaches are the most popular, simple and efficient methods for synthesizing the ZnO nanostructures. Carbon-black has a similar crystal structure to that of graphite but it is tridimensional and less ordered. The carbon-layers in carbon-black are parallel to each other but exhibit low order, frequently as concentric layers with turbostratic structures^20^. Carbon-black has high conductivity, large specific surface area, stability^21^, and a low cost, and it is naturally abundant^22^. Therefore, it has potential uses in carbon fillers^22^, reinforcing and support materials for metal catalysts^21^, Li-ion batteries^23^, biomaterials^24^, fuel cells^25^, photocatalysts^26^, solar cells^27^, oxygen-reduction electrocatalysts^28^, and rubber compounds^29^.

Heterostructures, including metal/semiconductor, semiconductor/metal, and semiconductor/semiconductor heterostructures, are useful for modifying the photoluminescence properties of semiconductors, in a manner determined by the reconfiguration of the band structure between the connecting materials in a steady state. Wang et al*.* reported that V_2_O_5_@Pt nanostructures exhibited improved photoluminescent intensity at λ = 466 nm^30^; Wang et al*.* reported that RGO@ZnO nanostructures exhibited enhanced intensity at near band edge emission^2^; Rajas-Lopez et al*.* found that MoS_2_/hBN/SiO_2_ exhibited enhanced photoluminescent intensity at the emission energy of 1.85 eV^31^; Chi et al*.* revealed that NiFe/ZnO exhibited photoluminescence with increased intensity at λ = 414 nm^32^; and Kandhasamy et al. found that MoS_2_/graphene exhibited enhanced photoluminescence at λ = 690 nm and 430 nm^33^. Based on the aforementioned properties of carbon-black, the potential uses of ZnO, and the useful properties of heterostructures, a sol–gel method and the thermal CVD process were used herein to fabricate ZnO/carbon-black heterostructures, and the effects of the carbon-black nanoparticle content on the ZnO crystal structure and photoluminescent properties were systematically investigated. The sample names for ZnO/carbon-black heterostructures are denoted as ZC0, ZC1, ZC2, ZC3, and ZC4, corresponding to the addition of carbon-black contents of 0, 2.08, 4.16, 6.25, and 8.33 × 10^−3^ mol, respectively. CB25 and CB500 represent the carbon-black nanoparticles before and after annealing at 500 °C, respectively.

## Results and discussion

### Analysis of crystal structure

**Figure 1:**
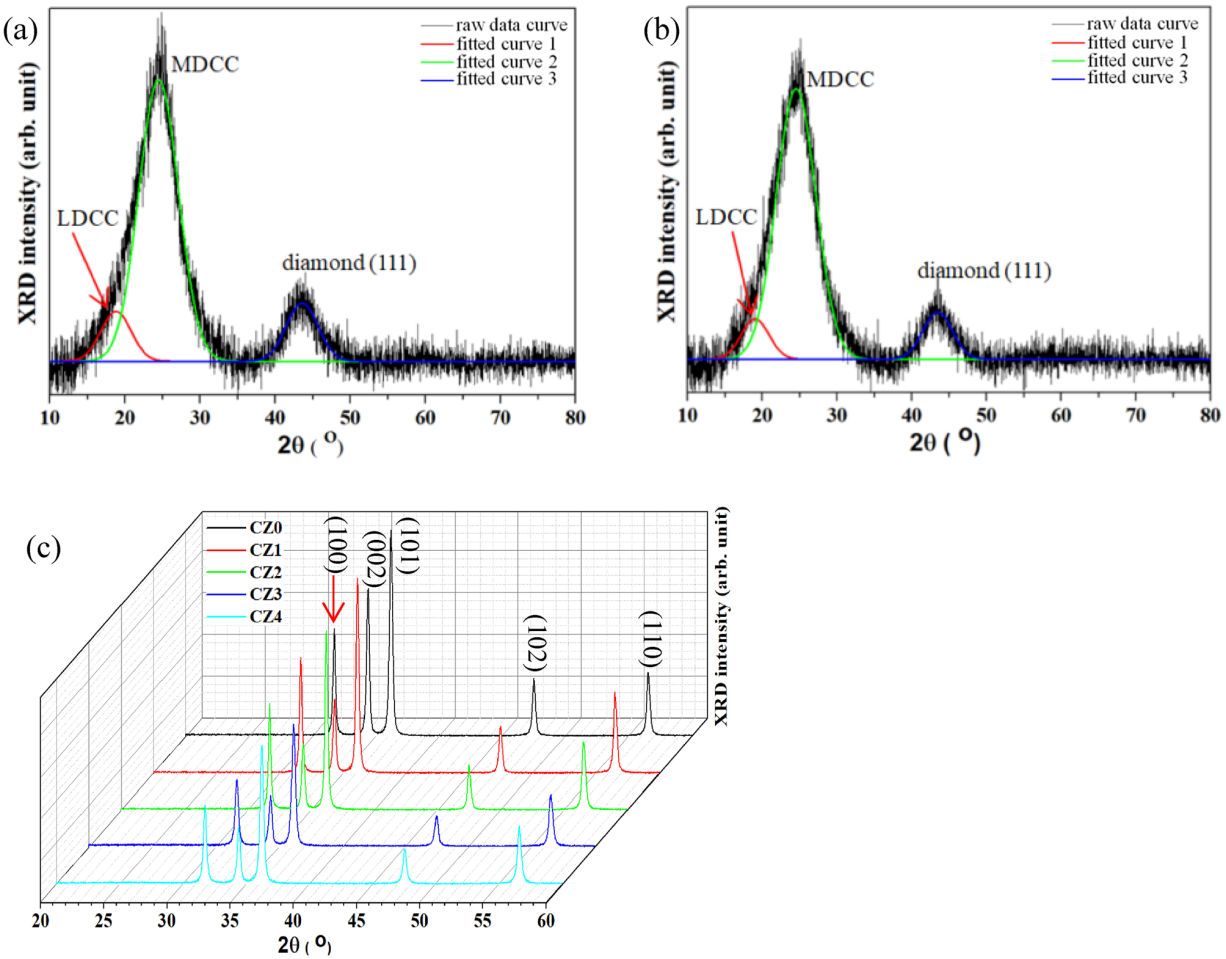
XRD patterns of carbon black treated at (**a**) 25 °C, (**b**) 500 °C, and (**c**) ZC NSs with various carbon-black contents.

Figure 1a,b presents the XRD patterns of the carbon-black NPs before and after annealing at 500 °C, respectively. Both raw data curves include an intense peak at around 24.1°, and a weak peak at around 43.5°. Crystalline graphite yields a peak at around 26.56°^34^, which is larger than that at approximately 2.46° obtained in this work. This result reveals that the micro-crystallinity of the carbon-black NPs differs from that of the graphite^34^. The shift in the XRD diffraction angle has the following causes; (1) impurity doping, (2) lattice distortion, (3) lattice shrinkage, (4) compressive stress (shifts toward higher angles), and (5) tensile stress (shifts toward lower angles). The impurity doping is not considered herein. Therefore, the shift in the diffraction angle is caused by the mechanical deformation as a result of distortion and stressing. Therefore, the more intense peak at 24.1° corresponds to the (002) plane of the graphite^34^ while the weaker one at 43.5° represents the (111) plane of the diamond^35^. The graphite phase has a higher electrical conductivity than the diamond phase. The ratio of the proportions of the graphite and diamond phases in the carbon-black are estimated as:

1 $$ \frac{{A_{graphite} }}{{A_{graphite} + A_{diamond} }} $$

and

2 $$ \frac{{A_{diamond} }}{{A_{graphite} + A_{diamond} }}, $$

respectively, where $A_{graphite}$ and $A_{diamond}$ are the integrated areas under the peaks of the graphite and diamond phases in the XRD patterns. The proportions of graphite (sp^2^-bonding) and diamond (sp^3^-bonding) in the carbon-black before thermal annealing at 500 °C are 87.2% and 12.8%, respectively, while those after annealing are 89.5% and 10.5%. These results confirm that the crystallinity of the graphite has been improved, so the thermal annealing improved the conductivity of the carbon-black. The peak of carbon (002) reveals that the carbon-black comprises the crystalline graphite phase and can be deconvoluted into the two peaks at around 19° and 24.4°. The former corresponds to the less-developed crystalline carbon (LDCC) phase and the latter corresponds to the more-developed crystalline carbon (MDCC) phase^34^. The respective intensity ratio I_MDCC_/I_LDCC_ are 5.7 (Fig. 1a) and 6.7 (Fig. 1b), implying that the crystallinity of the carbon-black was improved by annealing at 500 °C, favoring enhanced conductivity. Figure 1c includes XRD peaks of ZC NSs but no carbon-related peaks are observed. The significant peaks at around 31.7°, 34.4°, 36.2°, 47.5°, and 56.5° correspond to the ZnO(100), ZnO(002), ZnO(101), ZnO(102), and ZnO(110) planes, based on the JCPDS 36-1451^6^, confirming the typical ZnO hexagonal wurtzite structure. Table S1 presents the lattice constants, a (= b) and c, of ZnO and the c/a ratio. The lattice constants a and c, and the c/a ratio are around 0.325 and 0.52 nm, and 1.601, respectively. These results reveal that the carbon-black NPs have no effect on the crystal structure of the ZnO.

### Surface morphologies

**Figure 2:**
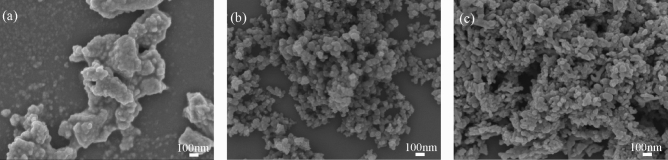
FESEM images of (**a**) ZC0, (**b**) carbon black, and (**c**) ZC4 NSs.

The surface morphologies of the carbon-black NPs, ZC0, and ZC4 NSs are obtained by the FESEM, as shown in Fig. 2a–c, respectively. The ZC0 NSs, as shown in Fig. 2a, are cluster-like NSs. Figure 2b reveals that the pure carbon-black NPs have particle-like morphologies. ZC4 NSs form particle-like clusters, as shown in Fig. 2c, similar to those in of the pure carbon-black NPs in Fig. 2b. The sol–gel procedure, without any capping agents, is the primary method used in this work to synthesize the ZC nanostructures. As a result, the nanostructures in ZC0 exhibit larger sizes, as shown in Fig. 2a. The smaller sizes of ZC4 NSs, as shown in Fig. 2c, can be attributed to the ZnO covering on the surface of the carbon-black nanoparticles (particles size ~ 26 nm). Additionally, for the FESEM analysis, the samples were initially suspended in deionized water, then dropped onto the silicon wafer surface, and finally dried under ambient conditions. Consequently, the densities of the nanostructures vary, as observed in Fig. 2a–c. Figure S1a exhibits an FESEM image of the ZC4 NSs which is circled by the red-line. Figure S1b displays the FESEM-EDX spectrum and presents the corresponding values of Zn, C, and O in the inset. Figure S1c–e presents the individual elemental mappings of Zn, C, and O in the ZC4 NSs. The mapping confirms the presence of Zn, C, and O. Most of the elemental Zn, C, and O are detected inside the red circle, as shown in Fig. S1c–e.

### Analysis of fine structure

**Figure 3:**
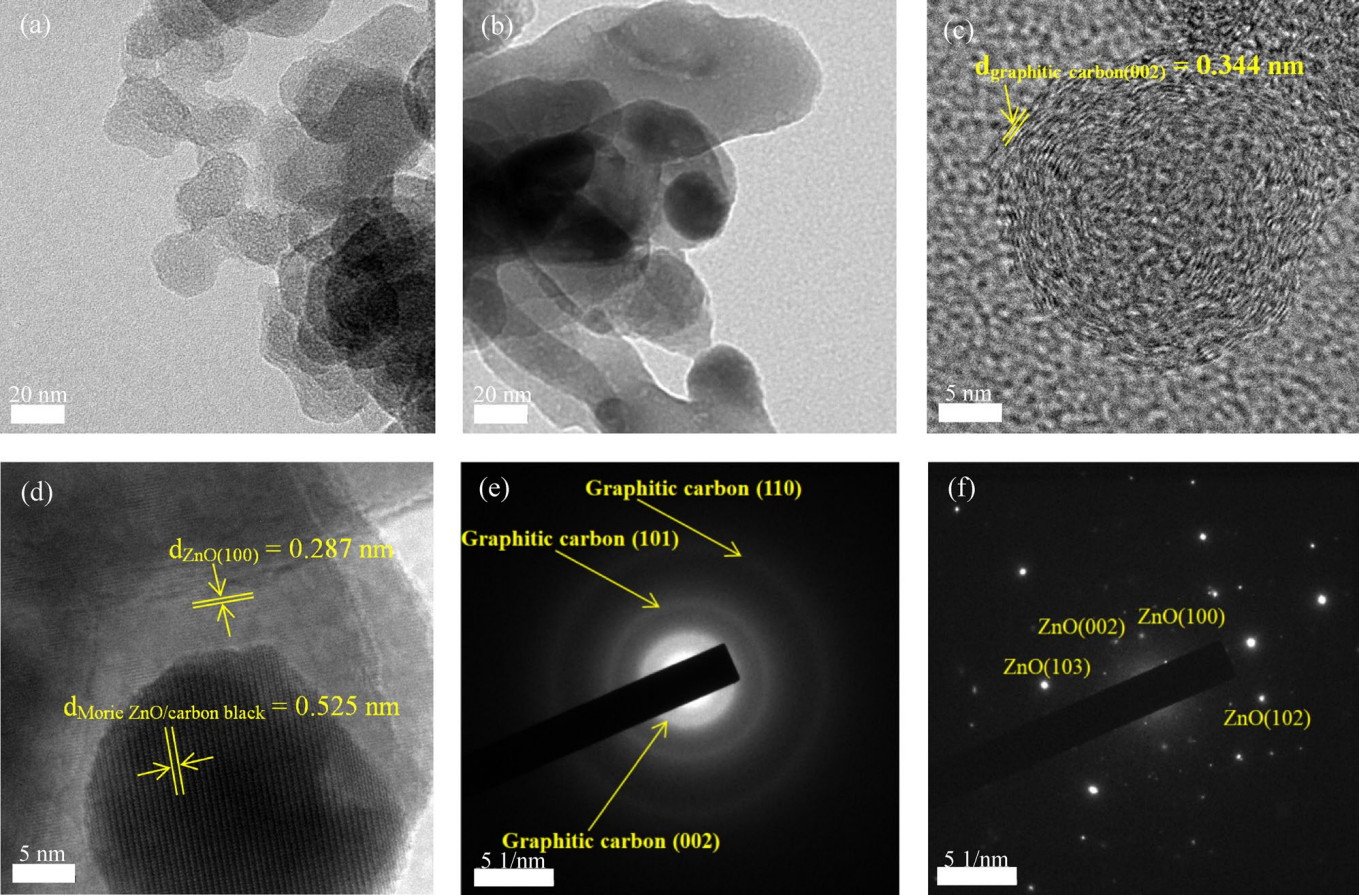
TEM images of (**a**) and (**b**), HRTEM images of (**c**) and (**d**), and SAD patterns of (**e**) and (**f**) of the carbon-black NPs and ZC4 NSs, respectively.

Figure 3a,c, and b,d present low-magnitude and HR-TEM images of the pure carbon-black NPs following the annealing at 500 °C and of the ZC4 NSs, respectively. The d-spacing corresponding to the graphitic carbon (002) plane is 0.344 nm, as shown in Fig. 3c. The selected area diffraction (SAD) pattern of the carbon-black NPs, as shown in Fig. 3e, reveals its poly-crystalline structure with the graphitic carbon (002), (101), and (110) d-spacings of 0.345, 0.204, and 0.116 nm, respectively^36^. These results confirm that the carbon-black NPs are crystalline, consistent with the XRD patterns as well. The HRTEM image of the ZC4 NSs, shown in Fig. 3d, presents ZnO (100) plane with a d-spacing of 0.287 nm. The obtained d-spacing of 0.525 nm does not correspond to the phase of ZnO or carbon-black. The increased d-spacing is associated with the Moiré pattern, which arises from the interaction between the lattices of ZnO and carbon-black. The SAD pattern of the ZC4 NSs, as shown in Fig. 3f, reveals the crystalline structure of the ZnO with ZnO (100), ZnO (002), ZnO (102), and ZnO (103) planes. These results reveal that the synthesized crystalline ZnO covered the surfaces of the carbon-black NPs.

### Analysis of the binding vibration

**Figure 4:**
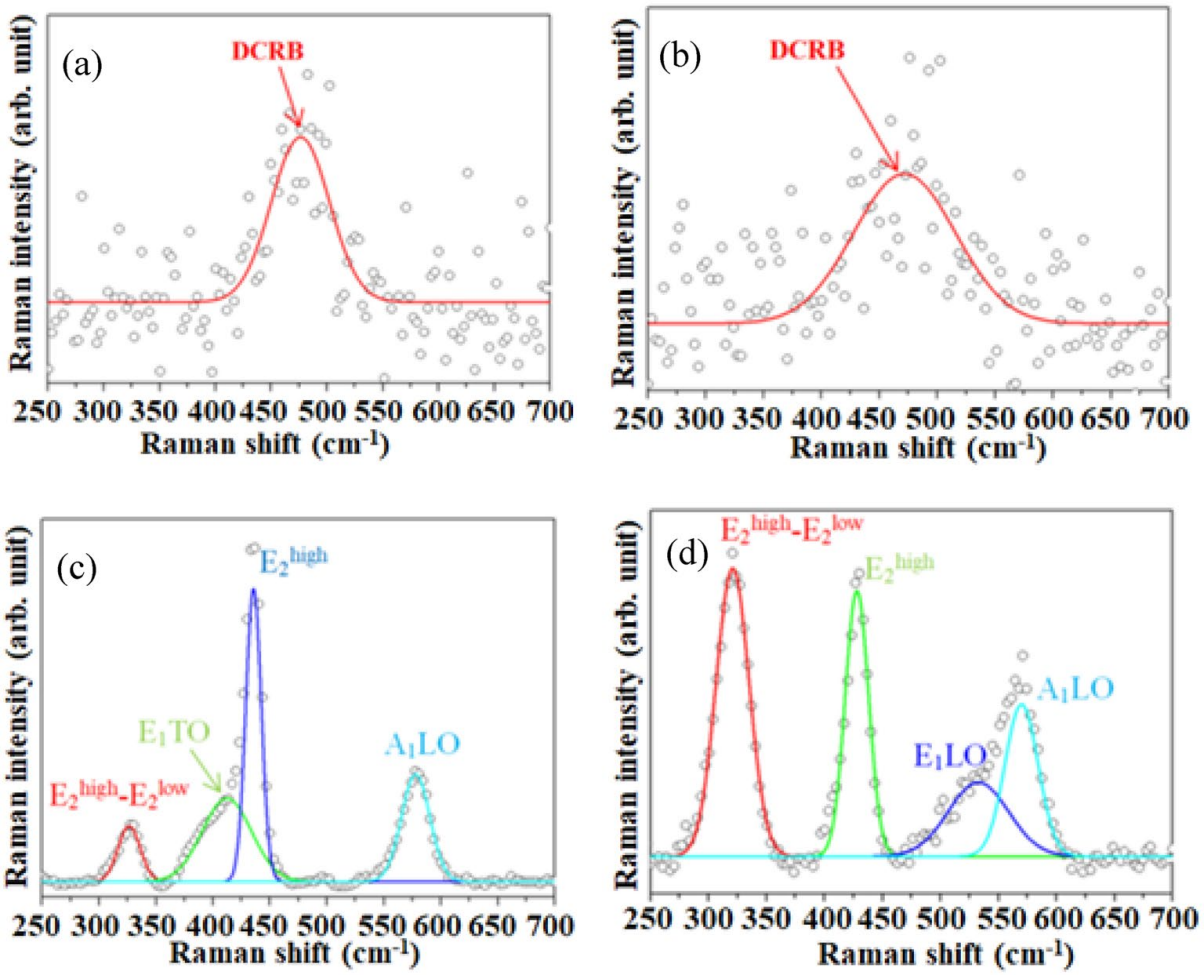
Raman spectra at the shorter Raman shifts of the (**a**) CB25, (**b**) CB500, (**c**) ZC0, and (**d**) ZC4 NSs.

Figure 4a–d present the Raman spectra of the carbon-black NPs before and after the annealing at 500 °C, ZC0 and ZC4 NSs, respectively. The peaks at around 476 and 471 cm^−1^ in Fig. 4a,b are attributed to the disordered carbon-related bond (DCRB)^37^. Figure 4c shows the Raman spectrum of ZC0, which includes four characteristic peaks of the wurtzite ZnO at 326, 412, 435, and 577 cm^−1^, corresponding to the E_2_^high^–E_2_^low^, E_1_TO, E_2_^high^, and A_1_LO modes, respectively^2^. E_2_^high^ is related to the oxygen in Zn–O in the wurtzite ZnO crystal structure^38^; A_1_LO is associated with oxygen deficiency in Zn–O^39^. A weak peak at 533 cm^−1^, shown in Fig. 4d, is attributable to the E_1_LO mode of wurtzite ZnO with the oxygen deficiency^40^. These results confirm that the hexagonal ZnO wurtzite structure has been established.

**Figure 5:**
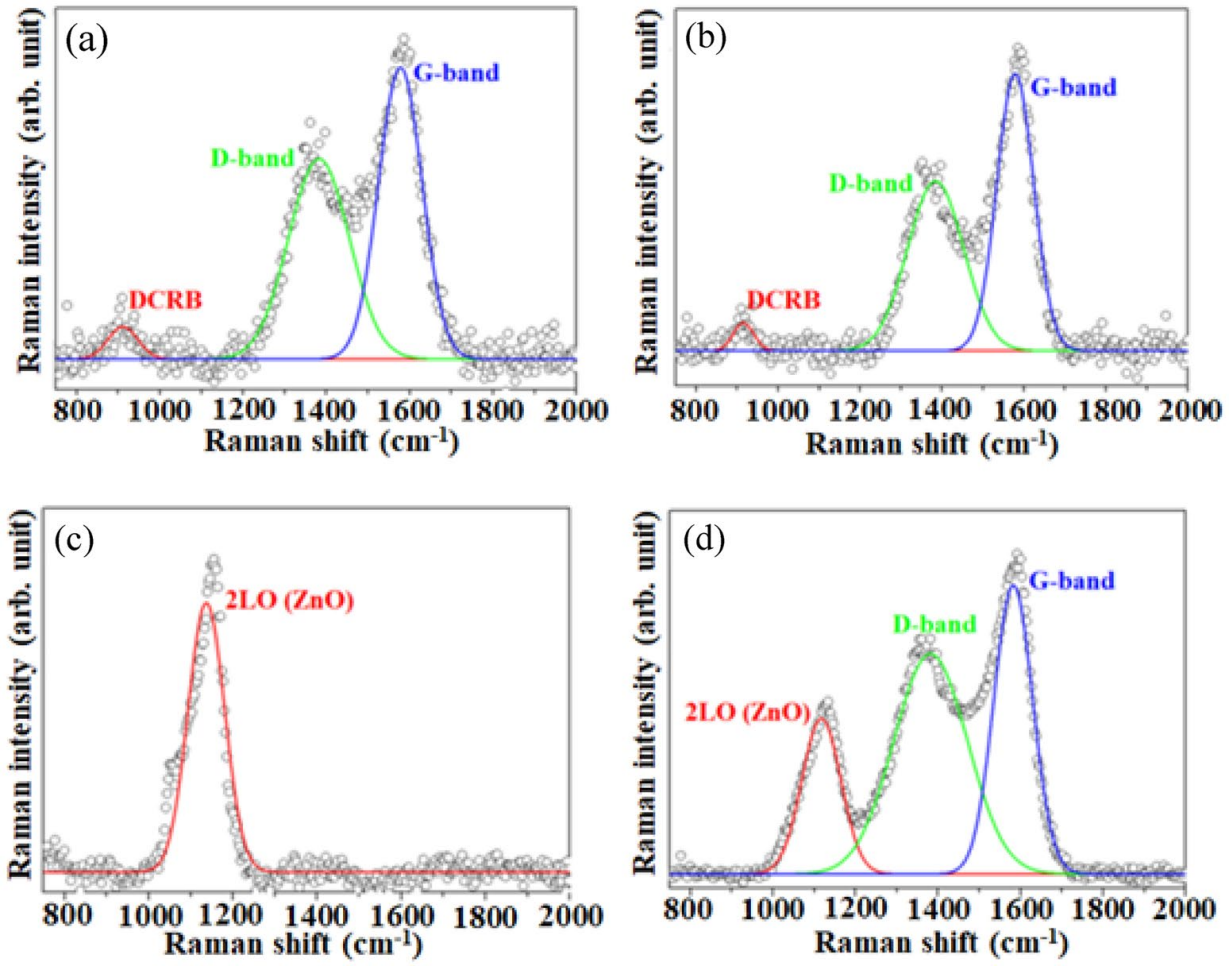
Raman spectra at the longer Raman shifts of the (**a**) CB25, (**b**) CB500, (**c**) ZC0, and (**d**) ZC4 NSs.

Figure 5a–d, show mainly the D- and G-bands of the carbon-based materials. Figure 5a,b present the spectra of the pure carbon-black NPs before and after the annealing at 500 °C, respectively, and include three significant peaks. The peak at around 910 cm^−1^ is attributed to the disordered carbon-related bonds^37^; those at 1382 cm^−1^ and 1577 cm^−1^ correspond to the D- and G-bands of the carbon-base materials. The former is associated with disordered sp^2^ carbon atoms while the latter is associated with the graphite-like sp^2^ carbon atoms^2^. The intensity ratios between the G-band and D-band in CB25 and CB500 are 1.633 and 1.457, respectively, as depicted in Fig. 5a,b. These findings suggest that the carbon-black nanoparticles exhibit improved graphitic carbon structures after annealing at 500 °C, which is consistent with the XRD results. Figure 5c shows a peak at 1136 cm^−1^, which is characteristic of the multiphonon 2LO vibration mode of the wurtzite ZnO crystal^41^. Figure 5d presents three peaks of the ZC4 NSs at 1117, 1381, and 1582 cm^−1^, which are associated with the vibration modes of ZnO 2LO, and carbon-based D- and G-bands, respectively. These results confirm the formation of the wurtzite ZnO crystal and the presence of carbon-black material in the ZC4 NSs.

### Absorption and optical band gap

**Figure 6:**
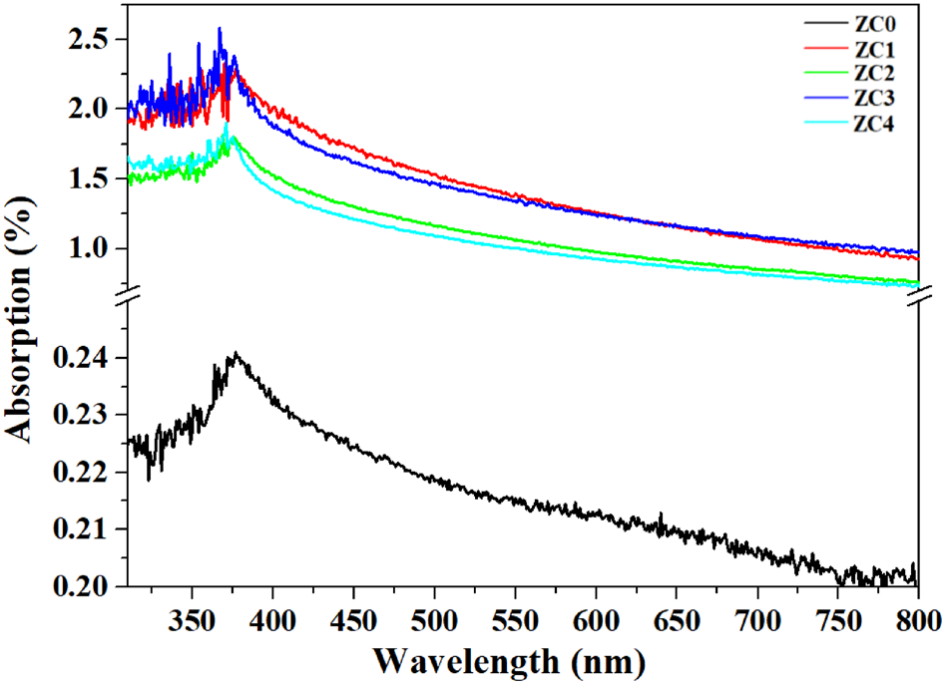
UV–visible absorption of the ZC NSs with various carbon-black NPs contents.

Figure 6 presents the absorptions by the ZC NSs, which were obtained using an integrating sphere. The absorption peak is located at the wavelength around 375‒400 nm, and the absorption by the ZC NSs is seven to ten times greater than that by the ZC0. The enhanced absorption by ZC NSs is attributable to the carbon-black NPs. Based on the absorption data, the optical band gaps of the ZC0, ZC1, ZC2, ZC3, and ZC4 NSs are estimated from the Tauc plot, as follows, and presented in Fig. S2a, S2b, S2c, S2d, and S2e, respectively.

3 $$ {\text{A}}(h\nu - E_{g} ) = (\alpha h\nu )^{n} $$

where *A* is a constant; *h* is Planck’s constant; *ν* is the frequency of the incident light; *n* is the characteristic constant of the material, and *E*_*g*_ is the band-gap of the material. The constant n is 2 for a direct *E*_*g*_ material, such as ZnO, and 1/2 for indirect *E*_*g*_ materials^42^. The values of *E*_*g*_ for ZC0 and ZC4 NSs that are estimated from the Tauc plot are 2.33 and 2.98 eV, respectively. Figure S2f shows the variation of the *E*_*g*_ of ZC NSs with increasing carbon-black NP contents. The crystallinity of graphite-like carbon is improved by annealing at 500 °C. The concentrations of free electrons that are transferred from carbon-black NPs to ZnO nanostructures increases with the carbon-black content. Therefore, the increase in *E*_*g*_ is caused by the Burstein–Moss effect.

### Photoluminescence properties

**Figure 7:**
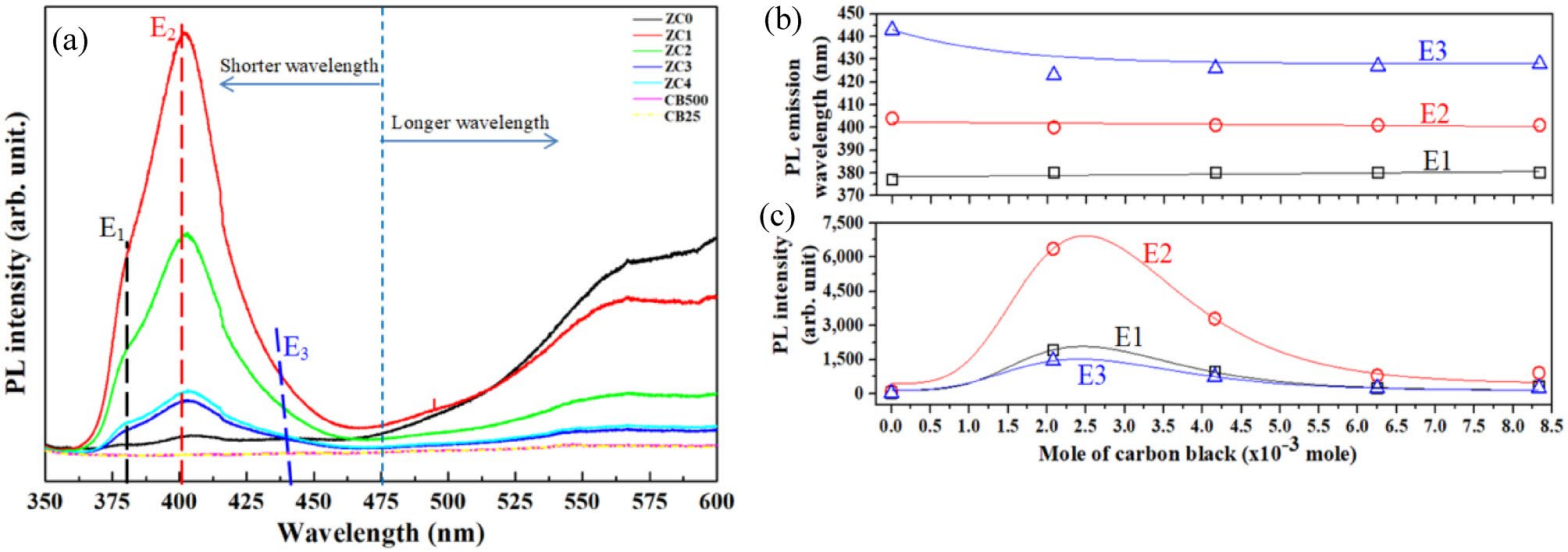
(**a**) PL spectra of ZC NSs with various carbon-black contents, (**b**) PL emission wavelengths and (**c**) PL intensities of ZC NSs with various carbon-black contents in the region of shorter wavelengths.

Figure 7a presents photoluminescence (PL) spectra of the pure carbon-black NPs and the ZC NSs. PL signals from the pure carbon-black NPs before and after annealing at 500 °C are not observed. The PL intensity of the ZnO, as shown in Fig. 7a, decreases at longer wavelengths (> 475 nm) as the carbon-black NP content increases. The PL intensity of the ZC NSs at shorter wavelengths (< 475 nm), as shown in Fig. 7a, increases with carbon-black NP content. Figure S3a–e present the deconvolutions of the PL spectra of the ZC0, ZC1, ZC2, ZC3, and ZC4 NSs, respectively, at short wavelengths (350‒475 nm). Three peaks are observed in such deconvoluted spectra. The peak at around 380 nm (E1, 3.26 eV) is attributable to the near band emission (NBE), which arises from the recombination of the electrons in the valence band (VB) of the ZnO and the holes in its conduction band (CB); the peak at 400 nm (E2, 3.1 eV) is associated with violet emission from the defect levels of the zinc interstitials $(Zn_{i}^{ \cdot \cdot } )$; the peak at 426‒440 nm (E3, 2.91‒2.81 eV) is attributable to transitions from the CB of the ZnO and the shallow donor-defect levels of $(Zn_{i}^{ \cdot \cdot } )$ to the acceptor defect levels of the zinc vacancies $(V_{Zn}^{{\prime\prime}} )$^2,43–45^.

Figure 7b shows that the wavelengths of PL emissions at E_1_ and E_2_ are not significantly shifted, while that at E_3_ is clearly blue-shifted. Figure S2f reveals that the optical band gaps of the ZC NSs increase with the carbon-black NP content, as a result of the Burstein–Moss effect. These results reveal that the electrons from the carbon-black NPs migrate mostly to the defect levels of the $(Zn_{i}^{ \cdot \cdot } )$. Figure 7c presents the PL intensities of the ZC NSs at E_1_, E_2_, and E_3_, respectively. The intensities of PL from ZC NSs at E_1_, E_2_, and E_3_ are enhanced by factors of 68.3, 62.8, and 56.8, respectively, when the carbon-black NP content is of the 2.03 × 10^−3^ mol. The PL intensity decreases as the carbon-back NP content is increased from 2.03 × 10^−3^ to 8.33 × 10^−3^ mol. Table S2 presents the PL intensities of the ZC NSs at short wavelengths and the relevant enhancement factors. The broad emission band at longer wavelengths (> 475 nm) is observed in Fig. 7a, and is identified with deep level emission (DLE). DLE arises from the defect levels within the band gap of the ZnO, such as oxygen vacancies $(V_{O}^{ \cdot \cdot } )$, zinc vacancies $(V_{Zn}^{{\prime\prime}} )$, zinc interstitials $(Zn_{i}^{ \cdot \cdot } )$, and oxygen antisites $(O_{Zn}^{\prime\prime\prime\prime} )$^8^. The defect levels of $(V_{O}^{ \cdot \cdot } )$ are primarily responsible for the DLE^46^. These results confirm that the proper carbon-black NP content incorporated increases PL intensity of ZnO in the short-wavelength region, and significantly reduces DLE. The related works on the photoluminescence properties of heterostructures comprising ZnO nanostructures and various carbon-related nanomaterials, such as reduced graphene oxides (RGO), carbon quantum dots (QDs), graphene, multiwall carbon nanotubes (MWCNTs), and carbon nanomaterials, are listed in Table S3. These studies indicate that the presence of carbon-related materials has an impact on the photoluminescence properties when integrated with ZnO nanostructures. Under appropriate conditions, the PL intensities of ZnO nanostructures can be enhanced through the incorporation of carbon-related materials. 

### Proposed band diagrams at shorter and longer wavelengths

**Figure 8:**
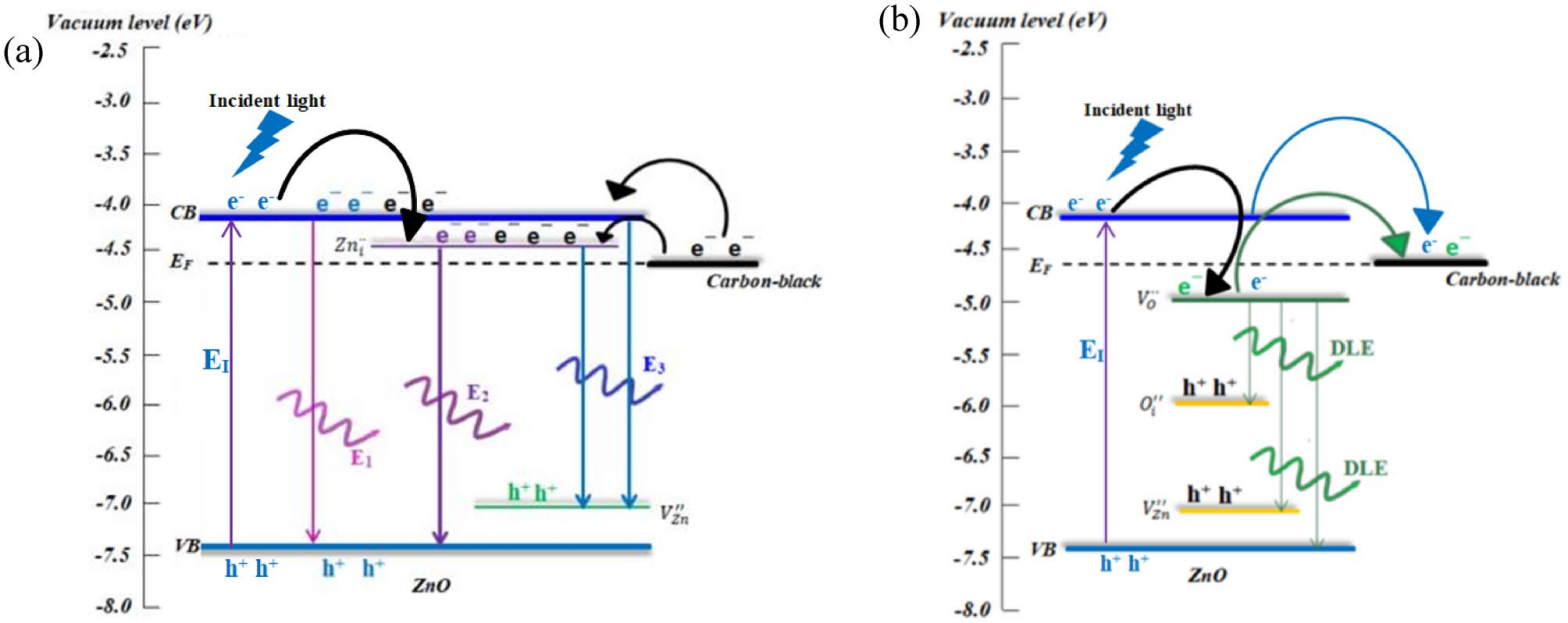
The proposed band diagrams of (**a**) ZC1 NSs at shorter and (**b**) ZC4 NSs at longer wavelength regions.

Figure 8a shows a proposed band diagram of the ZC1 NSs at short wavelength region. The electrons in the VB of the ZnO are excited to the CB by the incident light (E_I_). Some of these excited electrons should transfer to the lower defect level of $(Zn_{i}^{ \cdot \cdot } )$. In addition, the free electrons in the carbon-black NPs migrate to the CB of the ZnO and the shallow donor level of $(Zn_{i}^{ \cdot \cdot } )$ as a result of the improved conductivity of the carbon-black NPs and the enhanced electron transfer between ZnO and carbon-black NPs. Therefore, the emission intensities for ZC1 are increased^2,6^. Apart from the free electrons are form the CB of the ZnO to the $(Zn_{i}^{ \cdot \cdot } )$ defect level, the variations in the estimated optical band gap of the ZC NSs (Fig. S2f) show that the free electrons migrate mostly to the $(Zn_{i}^{ \cdot \cdot } )$ so the E_2_ emission is more intense than the E_1_ and E_3_ emissions. The PL intensities at shorter wavelength decrease as the carbon-black NPs content increases, as shown in Fig. 7a, which can be attributed to the following reasons. (a) the re-migration of the electrons from ZnO to the carbon-black NPs; (b) the high conducting carbon-black nanoparticles act as the carrier trapping sites, implying the increment of the free carriers trapping sites leading to the decreasing PL intensities. Figure 8b presents a proposed band diagram of the ZC4 NSs at longer wavelengths. The incident light (E_I_) excites the electrons from the VB of the ZnO to the CB. These excited electrons should transfer to the lower defect level of $(V_{O}^{ \cdot \cdot } )$ and migrate to carbon-black nanoparticles acting as the electron traps. The free electrons at the CB and the defect donor levels of $(V_{O}^{ \cdot \cdot } )$ migrate to the carbon-black NPs from the interface with ZnO as a result of the improved conduction of the carbon-black NPs. Therefore, the electrons migrate from the CB and $(V_{O}^{ \cdot \cdot } )$ defect level in the ZnO to the carbon-black nanoparticles leading to the decreasing DLE intensity by the ZC NSs^2,6^.

## Conclusions

The ZnO/carbon-black nanostructures were synthesized using the sol–gel method, and crystallized by annealing at 500 °C and 2 × 10^−2^ Torr. The crystal structures and bonding-vibration modes were systematically investigated and analyzed using the XRD, HRTEM, and Raman spectrometry. The Moiré patterns observed in the HRTEM images confirm that the carbon-black nanoparticles were covered by the ZnO crystals. The optical absorptance revealed that the band gap of the ZnO/carbon-black nanostructures increases with the carbon-black content owing to the Burstein–Moss effect. The photoluminescence spectra revealed that the emission intensities of the ZnO crystals at the near-band edge and in the violet and blue-light regions were enhanced by factors of 68.3, 62.8, and 56.8, respectively, when the carbon-black content was 2.03 × 10^−3^ mol. The proper carbon-black nanoparticle content increases the intensity of the luminescence of the ZnO crystals in the short-wavelength regime. The ZnO/carbon-black nanostructures have promises for use in the light-emitting devices.

## Materials and methods

### Preparation of pure ZnO and ZnO/carbon-black heterostructures

ZnO and the ZnO/carbon-black nanostructures (NSs) were fabricated using the sol–gel process from the precursor material zinc nitrate hexahydrate (Zn(NO_3_)_2_·6H_2_O, Choneye) that was mixed with carbon-black nanoparticles in DI water. A 0.28 M solution of Zn(NO_3_)_2_·6H_2_O in DI water was stirred for 60 min to ensure homogeneity. Then, highly conductive carbon-black nanoparticles with a size of 26 nm (NPs, Hondwen Co., Ltd.) were separately added in amounts of 0, 2.08, 4.16, 6.25, and 8.33 × 10^−3^ mol to the Zn-based solutions, which were stirred for 60 min. The aqueous mixtures thus obtained were baked in an oven for 30 h at 1 atm and 180 °C to remove moisture and yield dry mixed powder. Subsequently, the dried powder was put in a horizontal quartz-tube furnace and heated to 500 °C (10 °C/min), which was held for 10 min for crystallization at a pressure of 2 × 10^−2^ Torr. The final products were collected at room temperature (RT) and those with carbon-black contents of 0, 2.08, 4.16, 6.25, and 8.33 × 10^−3^ mol were denoted as ZC0, ZC1, ZC2, ZC3, and ZC4, respectively. The carbon-black nanoparticles before and after annealing at 500 °C are denoted as CB25 and CB500, respectively. Table 1 presents the precursor contents of zinc nitrate hexahydrate (Zn(NO_3_)_2_·6H_2_O) and carbon-black nanoparticles, as well as the annealing treatment of the samples.

**Table 1:** Relevant contents of precursors of zinc nitrate hexahydrate (Zn(NO_3_)_2_·6H_2_O), carbon-black nanoparticles, and subsequent annealing treatment of samples.

Sample name	Zn(NO_3_)_2_·6H_2_O (molar concentration)	Carbon-black nanoparticles (mole)	Annealing at 500 °C
CB25	0	4.3 × 10^−2^	No
CB500	0	4.3 × 10^−2^	Yes
ZC0	0.28	0	Yes
ZC1	0.28	2.08 × 10^−3^	Yes
ZC2	0.28	4.16 × 10^−3^	Yes
ZC3	0.28	6.25 × 10^−3^	Yes
ZC4	0.28	8.33 × 10^−3^	Yes

### Characterization of the pure ZnO and ZnO/carbon-black nanostructures

The crystal structures of ZC NSs were determined using a high-resolution transmission electron microscope (HRTEM; JEOL JEM-2010) and an X-ray diffractometer (XRD, Bruker D2 PHASER) with CuK_α_ radiation with λ = 1.5405 Å at 40 kV and 30 mA. Surface morphologies of ZC NSs were observed by the field emission scanning electron microscopy (FESEM; JOEL JSM-6335F). The bonding vibration modes of ZC NSs were analyzed by the Raman spectroscopy (3D Nanometer-scale Raman PL microspectrometer, Tokyo Instruments, INC.) with a semiconductor laser with an excitation wavelength of 488 nm. The absorption spectra of the ZC NSs in the UV–visible range were obtained using a UV–visible spectrophotometer (Cary 50, Varian). The photoluminescence (PL) spectra were obtained using a room-temperature confocal Raman spectrometer (Alpha 300, Witec, Germany) with a 266 nm semiconducting laser as the excitation source.

## Supplementary Information

Supplementary Information.

## Data Availability

The datasets used and/or analysed during the current study available from the corresponding author on reasonable request.

## References

[1] Alexandrov A, Zvaigzne M, Lypenko D, Nabiev I, Samokhvalov P (2020). Al-, Ga-, Mg-, or Li-doped zinc oxide nanoparticles as electron transport layers for quantum dot light-emitting diodes. Sci. Rep..

[2] Wang CC, Shieu FS, Shih HC (2020). Enhanced photodegradation by RGO/ZnO core-shell nanostructures. J. Environ. Chem. Eng..

[3] Tu Y, Kyle C, Luo H, Zhang DW, Das A, Briscoe J, Dunn S, Titirici MM, Krause S (2020). Ammonia gas sensor response of a vertical zinc oxide nanorods–gold junction diode at room temperature. ACS Sens..

[4] Liu X, Zheng Z, Wang J, Wang Y, Xu B, Zhang S, Hou J (2022). Fluidic manipulating of printable zinc oxide for flexible organic solar cells. Adv. Mater..

[5] Davis K, Yarbrough R, Froeschle M, White J, Rathnayake H (2019). Band gap engineered zinc oxide nanostructures via a sol–gel synthesis of solvent driven shape-controlled crystal growth. RSC Adv..

[6] Wang CC, Shieu FS, Shih HC (2021). Ag-nanoparticle enhanced photodegradation of ZnO nanostructures: Investigation using photoluminescence and ESR studies. J. Environ. Chem. Eng..

[7] Maldonado F, Stashans A (2010). Al-doped ZnO: Electronic, electrical and structural properties. J. Phys. Chem. Solids.

[8] Wang CC, Lin WC, Shieu FS, Shih HC (2019). Enhanced optoelectronic properties of thermally evaporated Sb-doped ZnO nanowires via defect structures. AIP Adv..

[9] Gupta H, Singh J, Dutt RN, Ojha S, Kar S, Kumar R, Reddy VR, Singh F (2019). Defect-induced photoluminescence from gallium-doped zinc oxide thin films: Influence of doping and energetic ion irradiation. Phys. Chem. Chem. Phys..

[10] Sajjad M, Ullah I, Khan MI, Khan J, Khan MY, Qureshi MT (2018). Structural and optical properties of pure and copper doped zinc oxide nanoparticles. Results Phys..

[11] Mazhdi M, Tafreshi MJ (2018). The effects of gadolinium doping on the structural, morphological, optical, and photoluminescence properties of zinc oxide nanoparticles prepared by co-precipitation method. Appl. Phys. A.

[12] Chen X, Xie Q, Li J (2020). Significantly improved photoluminescence properties of ZnO thin films by lithium doping. Ceram. Int..

[13] Gayathri S, Jayabal P, Kottaisamy M, Ramakrishnan V (2014). Synthesis of ZnO decorated graphene nanocomposite for enhanced photocatalytic properties. J. Appl. Phys..

[14] Chong SK, Dee CF, Rahman SA (2013). Structural and photoluminescence studies on catalytic growth of silicon/zinc oxide heterostructure nanowires. Nanoscale Res. Lett..

[15] Wang NW, Yang YH, Yang GW (2009). Indium oxide-zinc oxide nanosized heterostructure and whispering gallery mode luminescence emission. J. Phys. Chem. C.

[16] Khan SA, Khan T, Zulfiqar KM (2020). Enhanced photoluminescence performance of MoS_2_ nanostructures after amalgamation with ZnO NPs. Optik.

[17] Ahmadpour G, Nilforoushan MR, Boroujeny BS, Tayebi M, Jesmani SM (2022). Effect of substrate surface treatment on the hydrothermal synthesis of zinc oxide nanostructures. Ceram. Int..

[18] Wojnarowicz J, Chudoba T, Lojkowski W (2020). A review of microwave synthesis of zinc oxide nanomaterials: Reactants, process parameters and morphologies. Nanomaterials.

[19] Rashid TM, Nayef UM, Jabir MS, Mutlak FAH (2021). Synthesis and characterization of Au:ZnO (core:shell) nanoparticles via laser ablation. Optik.

[20] Martin-Martinez JM, Dillard DA, Pocius AV, Chaudhury M (2002). Adhesion science and engineering. Rubber Base Adhesives.

[21] Gautam RK, Verma A, Mohan SV, Varjani S, Pandey A (2019). Biomass, biofuels and biochemicals, microbial electrochemical technology. Electrocatalyst Materials for Oxygen Reduction Reaction in Microbial Fuel Cell.

[22] Tofighy MA, Mohammadi T, Yaragalla S, Mishra RK, Thomas S, Kalarikkal N, Maria HJ (2009). Carbon-based nanofillers and their rubber nanocomposites. Barrier, Diffusion, and Transport Properties of Rubber Nanocomposites Containing Carbon Nanofillers.

[23] Zhang L, Zhang M, Wang Y, Zhang Z, Kan G, Wang C, Zhong Z, Su F (2014). Graphitized porous carbon microspheres assembled with carbon black nanoparticles as improved anode materials in Li-ion batteries. J. Mater. Chem. A.

[24] Ianni ED, Jacobsen NR, Vogel UB, Møller P (2022). Systematic review on primary and secondary genotoxicity of carbon black nanoparticles in mammalian cells and animals. Mutat. Res. Rev. Mutat. Res..

[25] Fang B, Chaudhari NK, Kim MS, Kim JH, Yu JS (2009). Homogeneous deposition of platinum nanoparticles on carbon black for proton exchange membrane fuel cell. J. Am. Chem. Soc..

[26] Wen J, Li X, Li H, Ma S, He K, Xu Y, Fang Y, Liu W, Gao Q (2015). Enhanced visible-light H_2_ evolution of g-C_3_N_4_ photocatalysts via the synergetic effect of amorphous NiS and cheap metal-free carbon black nanoparticles as co-catalysts. Appl. Surf. Sci..

[27] Li P, Wu J, Lin J, Huang M, Huang Y, Li Q (2009). High-performance and low platinum loading Pt/Carbon black counter electrode for dye-sensitized solar cells. Sol. Energy.

[28] Song P, Zhang Y, Pan J, Zhuang L, Xu W (2015). Cheap carbon black-based high-performance electrocatalysts for oxygen reduction reaction. Chem. Commun..

[29] Farida E, Bukit N, Ginting EM, Bukit BF (2019). The effect of carbon black composition in natural rubber compound. Case Stud. Therm. Eng..

[30] Wang CC, Chen KC, Shieu FS, Shih HC (2019). Characterization and photoluminescence of V_2_O_5_@Pt core-shell nanostructures as fabricated by atomic layer deposition. Chem. Phys. Lett..

[31] Rojas-Lopez RR, Brant JC, Ramos MSO, Castro THLG, Guimarães MHD, Neves BRA, Guimarães PSS (2021). Photoluminescence and charge transfer in the prototypical 2D/3D semiconductor heterostructure MoS_2_/GaAs. Appl. Phys. Lett..

[32] Chi PW, Wei DH, Wu SH, Chen YY, Yao YD (2015). Photoluminescence and wettability control of NiFe/ZnO heterostructure bilayer films. RSC Adv..

[33] Kandhasamy DM, Mareeswaran PM, Chellappan S, Namasivayam D, Aldahish A, Chidambaram K (2022). Synthesis and photoluminescence properties of MoS_2_/graphene heterostructure by liquid-phase exfoliation. ACS Omega.

[34] Lee SM, Lee SH, Roh JS (2021). Analysis of activation process of carbon black based on structural parameters obtained by XRD analysis. Crystals.

[35] Sahu V, Shekhar S, Ahuja P, Gupta G, Singh SK, Sharma RK, Singha G (2013). Synthesis of hydrophilic carbon black; role of hydrophilicity in maintaining the hydration level and protonic conduction. RSC Adv..

[36] Dutta NJ, Mohanty SR, Buzarbaruah N (2016). Modification on graphite due to helium ion irradiation. Phys. Lett. A.

[37] Coccato A, Jehlicka J, Moens L, Vandenabeele P (2015). Raman spectroscopy for the investigation of carbon-based black pigments. J. Raman Spectrosc..

[38] Georgekutty R, Seery MK, Pillai SC (2008). A highly efficient Ag–ZnO photocatalyst: Synthesis, properties, and mechanism. J. Phys. Chem. C.

[39] Wu JJ, Liu SC (2002). Catalyst-free growth and characterization of ZnO nanorods. J. Phys. Chem. B.

[40] Marinho JZ, Romeiro FC, Lemos SCS, Motta FV, Riccardi CS, Li MS, Longo E, Lima RC (2012). Urea-based synthesis of zinc oxide nanostructures at low temperature. J. Nanomater..

[41] Romcevic N, Kostic R, Romcevic M, Hadzic B, Kuryliszyn-Kudelska I, Dobrowolski WD, Narkiewicz U, Sibera D (2010). Raman scattering from ZnO incorporating Fe nanoparticles: Vibrational modes and low-frequency acoustic modes. J. Alloys Compd..

[42] Arumugam J, Raj AD, Irudayaraj AA, Thambidurai M (2018). Solvothermal synthesis of Bi_2_S_3_ nanoparticles and nanorods towards solar cell application. Mater. Lett..

[43] Cao B, Cai W, Zeng H (2006). Temperature-dependent shifts of three emission bands for ZnO nanoneedle arrays. Appl. Phys. Lett..

[44] Raji R, Gopchandran KG (2017). ZnO nanostructures with tunable visible luminescence: Effects of kinetics of chemical reduction and annealing. J. Sci. Adv. Mater. Dev..

[45] Rao TP, Goswami GK, Nanda KK (2014). Detailed understanding of the excitation-intensity dependent photoluminescence of ZnO materials: Role of defects. J. Appl. Phys..

[46] Lin JH, Patil RA, Devan RS, Liu ZA, Wang YP, Ho CH, Liou Y, Ma YR (2014). Photoluminescence mechanisms of metallic Zn nanospheres, semiconducting ZnO nanoballoons, and metal-semiconductor Zn/ZnO nanospheres. Sci. Rep..

