# Supplementary material for: Zinc oxide nanostructures enhanced photoluminescence by carbon-black nanoparticles in Moiré heterostructures
Source: Sci Rep. 2023 Jun 15;13:9704. doi: 10.1038/s41598-023-36847-1 (PMC10272155; doi:10.1038/s41598-023-36847-1)
Supplement: Supplementary file 1 — Supplementary Information. [file 41598_2023_36847_MOESM1_ESM.docx]

| 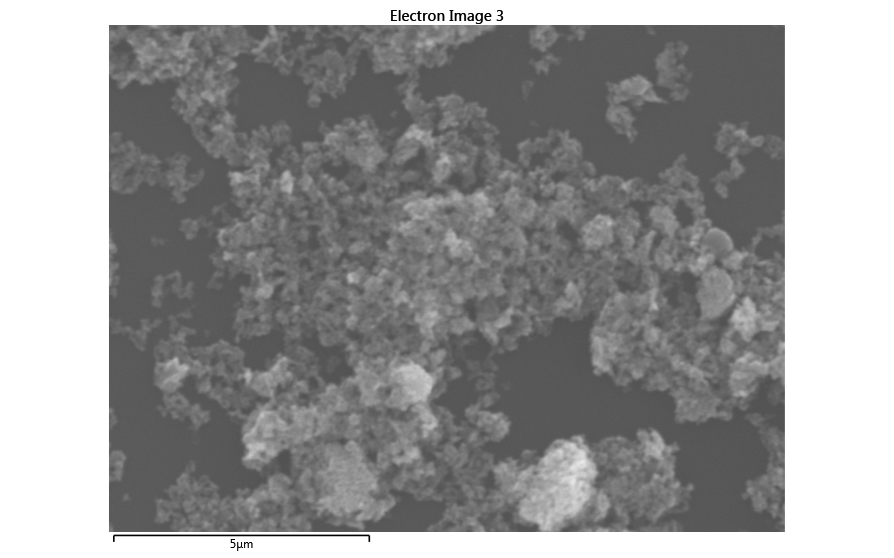  (a) | 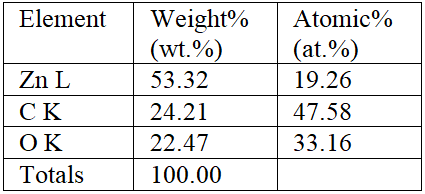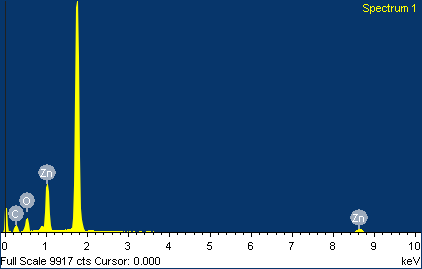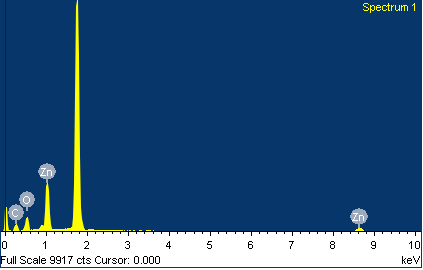  (b) |
| --- | --- |
| 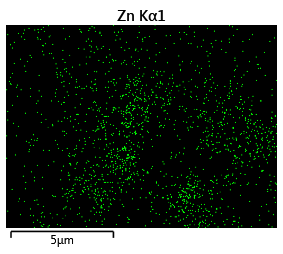  (c) | 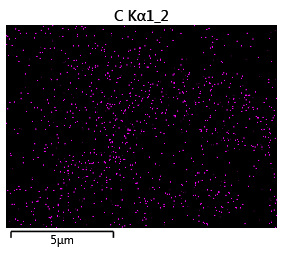  (d) |
| 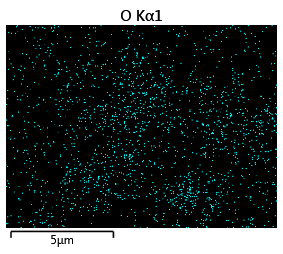  (e) |  |
| **Figure S1.** (a) FESEM images, (b) FESEM-EDX spectrum, and FESEM-mappings of the (c) Zn Kα1, (d) C Kα1, and (e) O Kα1 of ZC4 NSs. The inset in (b) shows the wt.% and at.% of the Zn, C, and O elements. | |

| 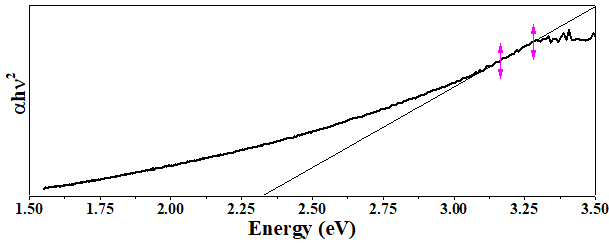  (a) | 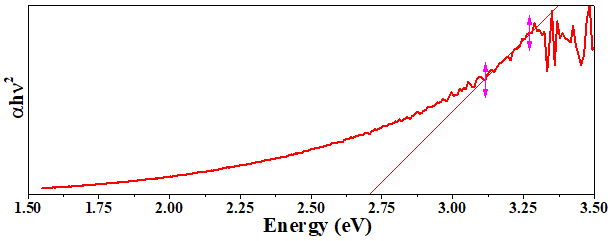  (b) |
| --- | --- |
| 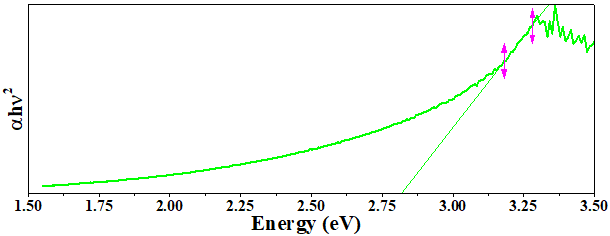  (c) | 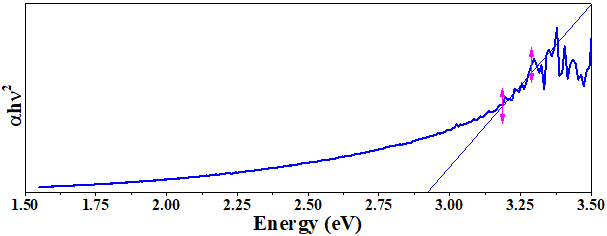  (f)  (0, 2.33)  (2.08, 2.71)  (4.16, 2.82)  (6.25, 2.92)  (8.33, 2.98)  (d) |
| 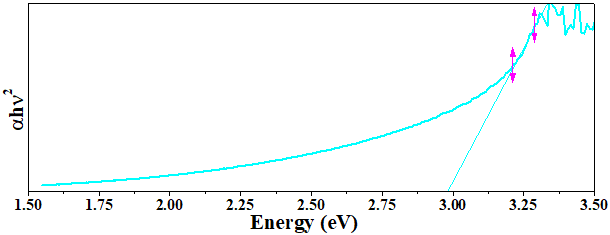  (e) | 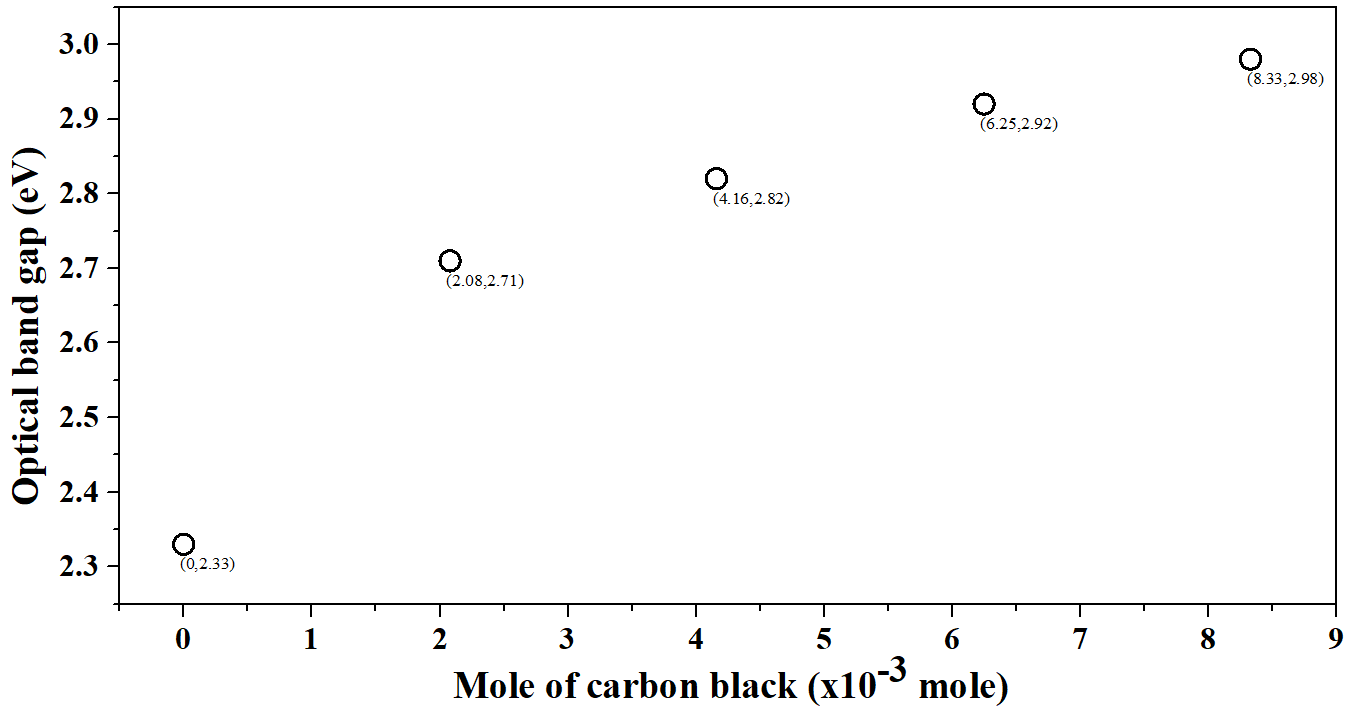 |
| **Figure S2.** The Tauc plots of (a) ZC0, (b) ZC1, (c) ZC2, (d) ZC3, and (e) ZC4 NSs. (f) optical band gaps with various carbon-black contents. | |

E2

E2

| 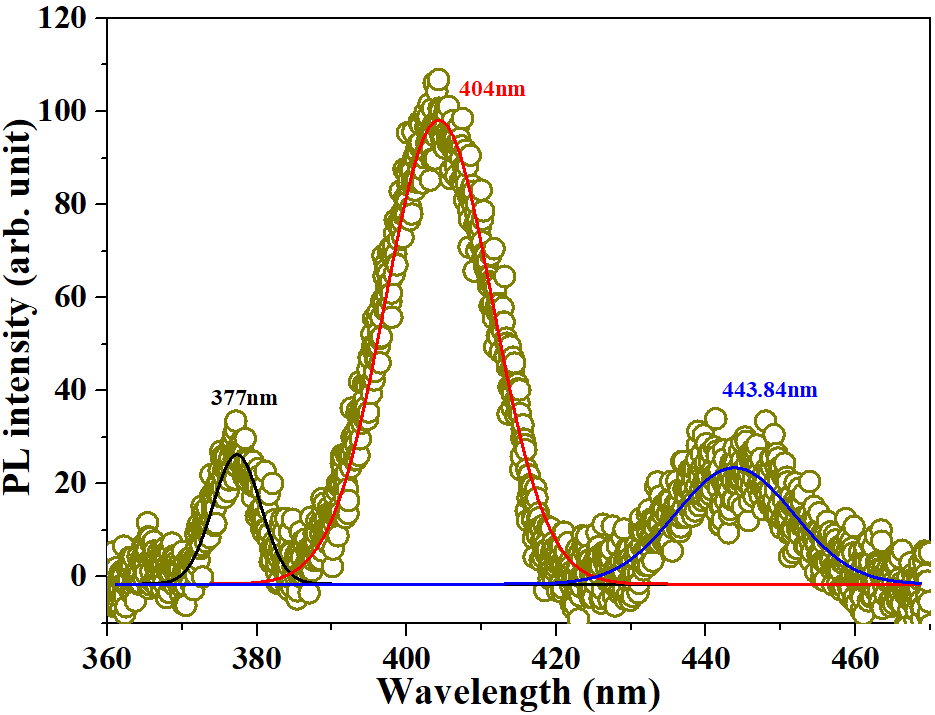  E3  E1  (a) | 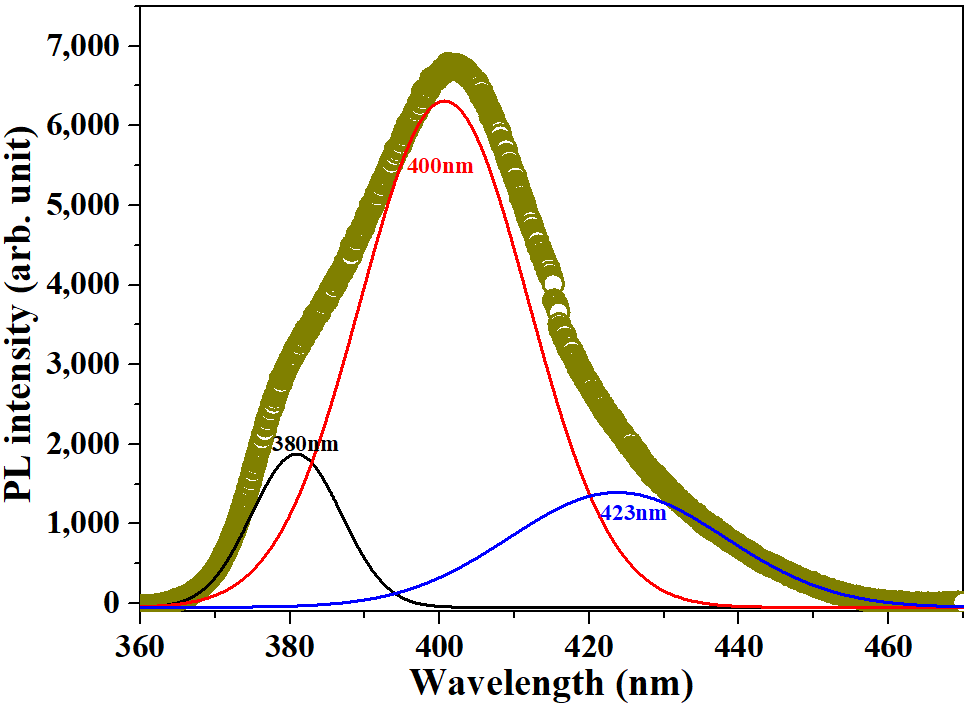  E3  E1  (b) |
| --- | --- |
| 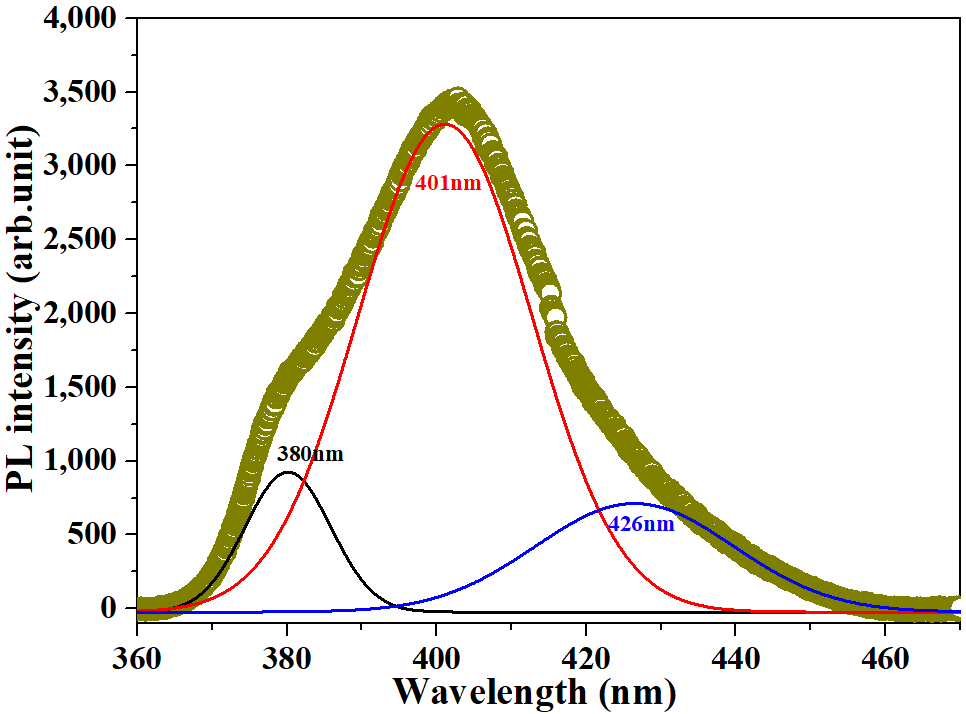  E1  E3  E2  (c) | 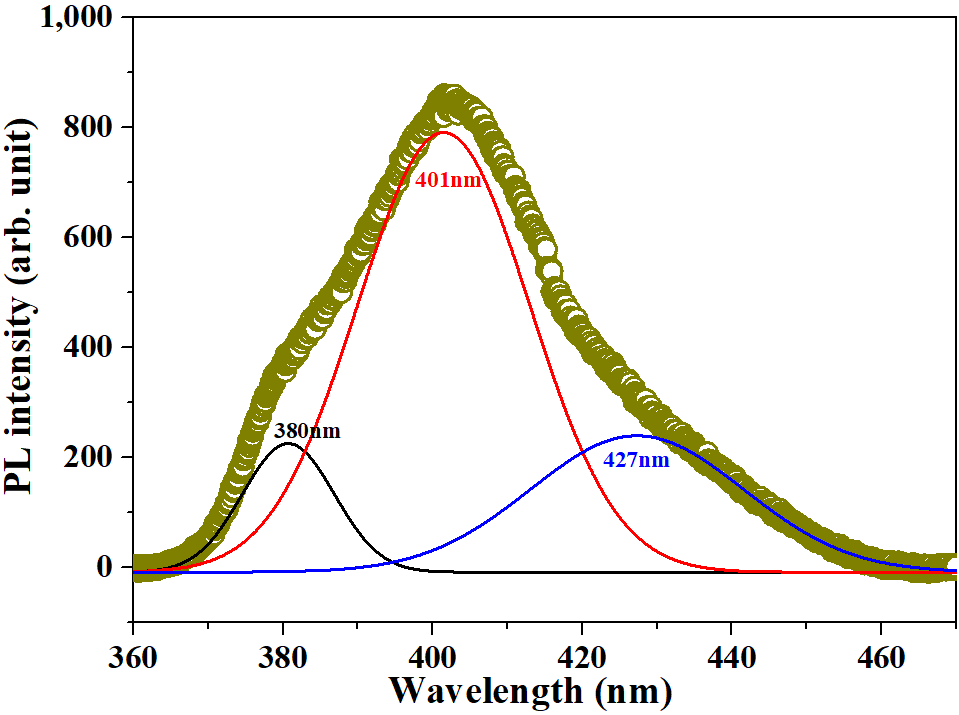  E3  E1  E2  (d) |
| 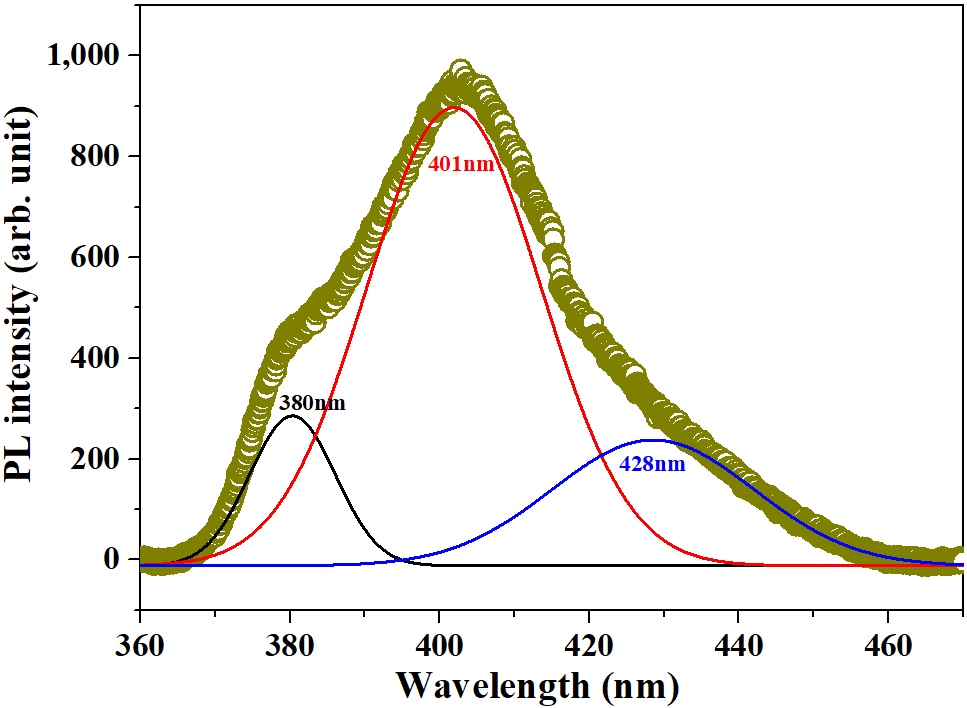  E3  E2  E1  (e) |  |
| **Figure S3.** The deconvolutions of the PL spectra of (a) ZC0, (b) ZC1, (c) ZC2, (d) ZC3, and (e) ZC4 NSs at the short wavelength region. | |

| **Table S1.** Lattice constants, a, b, c and c/a ratios of the ZnO crystal. | | | |
| --- | --- | --- | --- |
| **Sample** | **Lattice constants of ZnO (nm)** | | **c/a ratios** |
|  | **a (=b)** | **c** |  |
| **ZC0** | 0.3252 | 0.5208 | 1.6014 |
| **ZC1** | 0.3261 | 0.5221 | 1.6010 |
| **ZC2** | 0.3251 | 0.5208 | 1.6019 |
| **ZC3** | 0.3256 | 0.5215 | 1.6016 |
| **ZC4** | 0.3251 | 0.5207 | 1.6016 |

| **Table S2.** PL intensities and the enhancing factors of the ZC NSs at the range of the short wavelengths. | | | | | | |
| --- | --- | --- | --- | --- | --- | --- |
| **Samples** | **PL intensity (arb. unit)** | | | **Enhancing factor (%)** | | |
|  | **E1** | **E2** | **E3** | **E1** | **E2** | **E3** |
| **ZC0** | 27.77 | 99.63 | 24.96 | 0 | 0 | 0 |
| **ZC1** | 1924.71 | 6359.32 | 1443.2 | 6831 | 6283 | 5682 |
| **ZC2** | 948.08 | 3305.12 | 735.57 | 3314 | 3217 | 2847 |
| **ZC3** | 233.71 | 799.16 | 247.97 | 741 | 702 | 893 |
| **ZC4** | 297.3 | 908.47 | 249.39 | 971 | 811 | 899 |

| **Table S3**. Related studies on the photoluminescence properties of the heterostructures involving ZnO and carbon-related materials. | | | |
| --- | --- | --- | --- |
| Materials | Fabrication process | Results in PL properties | References |
| RGO/ZnO core-shell nanostructures | Sol-gel process | RGO/ZnO core-shell nanostructures exhibit increased intensities in the NBE emission (3.22 eV) and violet emission (3.1 eV) by factors of 10 and 5, respectively, as well as significantly decreased intensities in defect-related emission when compared to ZnO nanostructures. | [1] |
| Fe_3_O_4_@ZnO-C nanocomposites | Coprecipitation method | Fe_3_O_4_@ZnO-C composite enhances the intensities of UV and visible-light emission by approximately 8.8 and 3.65 times, respectively, compared to the Fe_3_O_4_@ZnO nanocomposites | [2] |
| ZnO/MWCNTs hybrid structures | MWCNTs: distributed electronic cyclotron resonance-plasma enhanced chemical vapour deposition  ZnO: RF magnetron sputtering | The ZnO/MWCNTs hybrid structures enhance the band edge emission (390 nm) by a factor of 3 compared to the ZnO thin films. | [3] |
| C/ZnO hybridized quantum dots (QDs) | Carbon dots: chemical process C/ZnO hybridized QDs: chemical process | C/ZnO QDs increase the PL intensities at 440 and 531 nm by factors of 1.53 and 2.08, respectively, when compared to pristine carbon QDs and ZnO QDs. | [4] |
| C@ZnO core-shell nanostructures | Hydrothermal carbonization process | The C@ZnO core-shell nanostructures exhibit an increased intensity ratio between the NBE emission (393 nm) and the defect-related emission (523 nm) by several folds compared to the ZnO nanorods. | [5] |
| ZnO/graphene hybrids | Electrophoretic deposition and magnetron sputtering technique | ZnO/graphene hybrids show the decrease PL intensities in the regions of blue emission (~400 nm) and defect-related emission (visible light region) by several folds compared to pure ZnO nanostructures. | [6] |
| Carbon quantum dots/ZnO heterostructures | Hydrothermal method | The carbon quantum dots/ZnO heterostructures present a decreased intensities at 425 and 480 nm by several folds when compared to the pristine ZnO microspheres | [7] |
| ZnO-carbon nanocomposites | Chemical precipitation method | The ZnO-carbon nanocomposites demonstrate a decrease in PL intensities across the range of 350 to 600 nm compared to the pristine ZnO nanostructures. | [8] |
| ZnO:MWCNTs nanocomposites | Chemical method | The PL intensities at NBE and DLE are enhanced by factors of 6 and 1.5, respectively, in the ZnO:MWCNTs heterostructures. | [9] |
| ZnO/carbon-black heterostructures | Sol-gel process | PL intensities at wavelengths around 380, 400, and  430nm are enhanced by factors of 68.3, 62.8, and 56.8,  respectively, when the ZnO nanostructures are hybridized  with the carbon-black nanoparticles. | This work |

**References**

1. Wang CC, Shieu FS, Shih HC (2020) Enhanced photodegradation by RGO/ZnO core-shell nanostructures. *J. Environ. Chem. Eng.* 8:103589.
2. Arief AS, Muldarisnur M, Zulhadjri, Usna SRA (2023) Enhancement in photoluminescence performance of carbon-based Fe_3_O_4_@ZnO-C nanocomposites. *Vacuum* 211: 111935.
3. Ouldhamadouche N, Achour A, Musa I, Aissa KA, Massuyeau F, Jouan PY, Kechouane M, Brizoual LL, Faulques E, Barreau N, Djouadi MA (2012) Structural and photoluminescence characterization of vertically aligned multiwalled carbon nanotubes coated with ZnO by magnetron sputtering. *Thin Solid Films* 520: 4816-4819.
4. He L, Mei S, Chen Q, Zhang W, Zhang J, Zhu J, Chen G, Guo R (2016) Two-step synthesis of highly emissive C/ZnO hybridized quantum dots with a broad visible photoluminescence. *Appl. Surf. Sci.* 364: 710-717.
5. Chen T, Yu S, Fang X, Huang H, Li L, Wang X, Wang H (2016) Enhanced photocatalytic activity of C@ZnO core-shell nanostructures and its photoluminescence property. *Appl. Surf. Sci.* 389: 303-310.
6. Ding J, Yan X, Xue Q (2012) Study on ﬁeld emission and photoluminescence properties of ZnO/graphene hybrids grown on Si substrates. *Mater. Chem. Phys.* 133: 405-409.
7. Wang L, Li X, Jin Y, Liu G, Shan Y (2023) Integrating photoluminescence and ferromagnetism in carbon quantum dot/ZnO by interfacial orbital hybridization for multifunctional bioprobes. *Chem. Phys. Chem.* 24: e202200766.
8. Babar SB, Gavade NL, Bhopate DP, Kadam AN, Kokane SB, Sartale SD, Gophane A, Garadkar KM, Bhuse VM (2019) An efficient fabrication of ZnO–carbon nanocomposites with enhanced photocatalytic activity and superior photostability. *J. Mater. Sci. Mater. Electron.* 30: 1133-1147.
9. Rauwel E, Galeckas A, Rosario Soares M, Rauwel P (2017) Inﬂuence of the interface on the photoluminescence properties in ZnO carbon-based nanohybrids. *J. Phys. Chem. C* 121: 14879−14887.
